# Active Transport and Health Outcomes: Findings from a Population Study in Jiangsu, China

**DOI:** 10.1155/2013/624194

**Published:** 2013-04-04

**Authors:** Shu-rong Lu, Jian Su, Quan-yong Xiang, Feng-yun Zhang, Ming Wu

**Affiliations:** Chronic Disease Department of Jiangsu Provincial Center for Disease Control and Prevention, 172 Jiangsu Road, Nanjing 210009, China

## Abstract

To investigate the prevalence of active transport (AT, defined as walking or bicycling for transport) and to explore the association between AT and health outcomes, we conducted a population-based cross-sectional study in Jiangsu, China, where walking and bicycling are still the main modes of transport. In this study, 8400 community residents aged 18 or above were interviewed following a multistage random sampling method (100% response rate). Face-to-face questionnaire survey data, anthropometric measurements, and biochemical data from blood tests were collected. Results show that 49.6% of the subjects, as part of daily transport, actively traveled on average 5.3 days per week, 53.5 minutes per day, and 300.3 minutes per week. There was an inverse correlation between AT and some health outcomes: AT respondents had a higher prevalence of cholesterol disorder; AT respondents who actively travelled every day had a higher risk of diabetes, whilst AT respondents with shorter daily or weekly duration had a lower risk of obesity, central obesity, and cholesterol disorder. Moreover, AT influences more health aspects among urban residents than among rural residents. Findings of this study do not support the notion that AT is beneficial to population health. Further research is needed in determining the negative side effects of AT.

## 1. Introduction

As the result of rapid economic development in recent years, many countries are experiencing a fast-growing automobile population and steadily improving public transportation infrastructure, which accelerate the transition from active to passive transport. Active transport (AT), which usually means walking, cycling, and using public transport, could have multiple health benefits by increasing physical activity, protecting people from some chronic diseases and reducing the adverse health effects of motor vehicle transport [1–3]. Many countries strongly promote a population shift from car dependency to active transport [4, 5]. In 2008, in order to promote a healthier lifestyle, the Chinese government launched a nationwide program, of which AT is an integral part [6]. However, some studies have reported that AT was related to no or even negative effects on health [7, 8].

As being the largest developing country, China has her unique characteristics concerning transport environment and residents' transport activity. However, there is little data on these aspects available at present. Jiangsu Province is one of the most economically booming areas in the southeast of China. With a population of 78.66 million, Jiangsu is witnessing the transition from AT transport mode to increased car dependency. In this study, we investigated the prevalence, frequency, and duration of AT and explored the relationship between AT and health outcomes at a population level.

## 2. Materials and Methods

### 2.1. Study Design

Jiangsu Provincial Surveillance (JPS) on chronic diseases and behavioral risk factors is a population-based cross-sectional survey carried out in every 3 years in 14 disease surveillance points (DSPs, in the unit of county) of Jiangsu Province, China. These 14 DSPs are spread across all municipal cities of Jiangsu and were shown to be representative of Jiangsu for geographic coverage and size, population, death rates, economic development level, and other characteristics [9]. In this paper, we analyzed AT data of the latest JPS carried out between August and November of 2010.

### 2.2. Sampling

**Figure 1:**
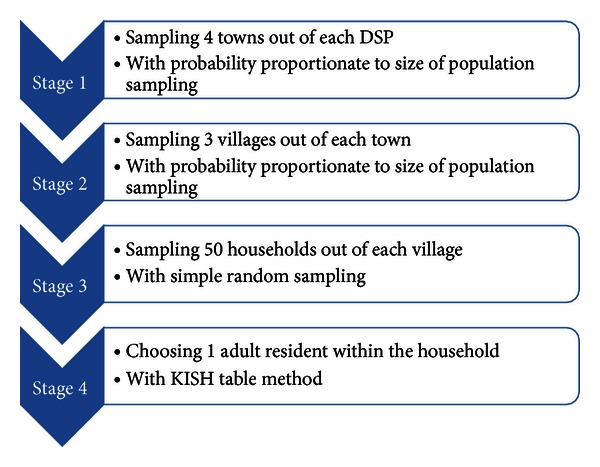
Sample assignment and sampling methods.

A multistage stratified sampling method (see Figure 1) was employed. 8400 community residents aged 18 or above were selected. The response rate was 100%.

Written consent was obtained from all the participants.

### 2.3. Data Collection

- Face-to-face interviews using a structured questionnaire were conducted by trained public health practitioners. Information on frequency and duration of active transport within one typical week was collected. To assess the overall physical activity level, strength, frequency and duration of occupation, and exercise-related physical activities within one typical week were also recorded. Information on demographics and self-reported health was collected simultaneously.
- Anthropometric measures on body weight were collected using an electronic scale (TANITA HD-390), height, and waist circumstance (WC) with soft tape. Systolic blood pressure (SBP) and diastolic blood pressure (DBP) were also measured by electronic blood meter (OMRON HEM-7071) three times, and the average was calculated.
- Biochemical tests using fasting blood were conducted for glucose (FBG) and lipemia, including total cholesterol (TC), high-density lipoprotein cholesterol (HDL-C), low-density lipoprotein cholesterol (LDL-C), and triglycerides (TG).

### 2.4. Study Variables

*Active Transport (AT).* Walking and/or bicycling for transport was accessed by the question “Do you usually have more than 10 minutes' walking and/or bicycling (includes both traditional and electric) in transport?” Walking or bicycling for occupational reasons, couriers' walking or bicycling during work time, for example, or for exercise was not included. 

*Obesity.* Body mass index (BMI = body weight/height^2^) ⩾ 24 kg/m^2^ [10].

*Central Obesity.* WC of male ⩾ 90 cm or WC of female ⩾ 80 cm [11].

*Hypertension.* Average of thrice SBP ⩾ 140 mmHg and/or average of thrice DBP ⩾ 90 mmHg [12].

*Diabetes.* FBG ⩾ 7.0 mmol/L [13].

*Cholesterol Disorder.* TC ⩾ 6.22 mmol/L and/or HDL-C < 1.04 mmol/L and/or LDL-C ⩾ 4.14 mmol/L and/or TG ⩾ 2.26 mmol/L [14].

*Overall Physical Activity Level*. It was categorized into “active,” “moderate,” and “inactive” according to frequency and duration, total amount of transport, occupation, and exercise-related physical activity within one typical week [15]. “Active” was developed to describe higher levels of participation, which equates to approximately at least one hour per day or more, of at least moderate-intensity activity above the basal level of physical activity; “moderate” is proposed as a level of activity equivalent to half an hour of at least moderate-intensity PA on most days; “inactive” is simply defined as not meeting any of the criteria for either of the previous categories.

*Educational Level Category.* Illiterate or finishing primary education as “low,” finishing university or above education as “high,” all others as “medium.”

*Household Income Level Category.* Lower than the *Q*_25_ of household income as “low,” upper than the *Q*_75_ as “high,” and all others as “medium.”

### 2.5. Statistical Analysis

Survey responses were entered into EpiData3.1 with double-check method. All the analyses in this paper were performed with SPSS Statistics 19.0. Independent-samples *t*-test, one-way ANOVA, and Chi-square tests were used to compare differences in variables. The association was analyzed using logistic regression models, 0 for non-AT or no-disease, 1 for AT or disease, 0.05 for probability of entry, and 0.10 for removal. Statistical significance was considered when *P* < 0.05 (two sided).

## 3. Results

Females account for 54.7% of all the 8400 participants aged 18–94 (mean age: 52.5 years). AT respondents are significantly different to non-AT respondents in the composition of gender, age group, educational level, and household income level. 

### 3.1. Prevalence, Frequency, and Duration of AT

#### 3.1.1. Prevalence

The prevalence of AT was 49.6% (*n* = 4166) in general, while it was higher among females (52.8%), the middle-aged or the aged (57.0% for 55–64 age group and 55.3% for 65 or above age group), people of low or high educational level (53.0%), and people of low household income level (54.4%). Results of Chi-square test showed that, compared with non-AT respondents, AT respondents have lower percentages of inactive level of physical activity and higher percentages of active level of physical activity. There is no significant difference between urban residents (49.2%) and rural residents (49.8%) on the prevalence of AT. The prevalence of AT among different groups of subjects was shown in Table 1.

#### 3.1.2. Frequency and Duration of AT

For the 4166 subjects with AT, on average, they actively traveled for transport on 5.3 days per week, for 53.5 minutes per day, and 300.3 minutes per week. Males had more days (5.4) and longer duration (54.2 min/day, 308.6 min/week) of AT than those of females. Compared with rural residents, urban residents were more frequently on AT (5.5 days/week, *P* < 0.01) but with shorter daily duration (48.5 minutes, *P* < 0.01). On the whole, both daily duration and weekly duration of AT increased with age (59.9 min/day and 340.7 min/week for 65 or above age group). With the increase of household income level, AT also increased in frequency but with shorter duration. Daily and weekly duration of AT decreased with the increase of educational level as well (data not shown). 

### 3.2. Health Outcomes Difference with AT

#### 3.2.1. Health Outcomes Difference between AT and Non-AT Respondents

Compared with non-AT respondents, subjects with AT had a higher prevalence of central obesity, hypertension, and cholesterol disorder. Through further analysis of health indices, we found that the difference in the prevalence of hypertension mainly comes from SBP, while difference in the prevalence of cholesterol disorder is mainly from TC and HDL-C. Differences on prevalence of chronic disease and means of health indices between AT and non-AT respondents are shown in Table 2.

#### 3.2.2. Health Outcomes/Indices with Frequency and Duration of AT

Among AT responds, the prevalence of hypertension and average level of WC increased with both frequency and daily duration of AT (*P* < 0.05). Meanwhile, the prevalence of cholesterol disorder and average level of SBP and HDL-C increased with daily duration of AT (*P* < 0.05). The prevalence of health outcomes/the mean level of health indices among different groups of AT frequency and AT duration were shown in Table 3.

### 3.3. Multinomial Analysis

#### 3.3.1. Association between AT and Health Outcomes

Results of the logistic regression models show that, after being adjusted for age, gender, educational level, household income level, and overall physical activity level, the odds ratio (OR) of cholesterol disorder for AT respondents compared with non-AT respondents is 1.26 (95% C.I 1.13–1.41, *P* < 0.01). The association between central obesity/hypertension and AT disappeared after adjustment in the logistic regression models (see Table 4).

#### 3.3.2. Association between Frequency, Daily Duration, Weekly Duration of AT, and Health Outcomes

Taking frequency and daily duration of AT into logistic regression simultaneously, results reveal that, among AT respondents, people who have AT 3–6 days per week have lower risk of diabetes than those who have AT every day (OR = 0.73, 95% CI 0.53–0.92, *P* < 0.05). Between people who have AT less than 30 minutes per day and people who have AT more than 60 minutes per day, the ORs of obesity, central obesity, and cholesterol disorder are 0.63 (95% CI 0.48–0.83, *P* < 0.01), 0.82 (95% CI 0.68–0.99, *P* < 0.05), and 0.68 (95% CI 0.56–0.82, *P* < 0.01), respectively (see Table 4). By analyzing urban residents and rural residents separately, we found that, among urban residents, less frequent AT was associated with lower prevalence of central obesity (OR = 0.66, *P* < 0.05), diabetes (OR = 0.57, *P* < 0.05), and cholesterol disorder (OR = 0.67, *P* < 0.01); shorter daily duration of AT was associated with lower prevalence of central obesity (OR = 0.67, *P* < 0.01), obesity (OR = 0.61, *P* < 0.05), and cholesterol disorder (OR = 0.61, *P* < 0.01) whereas, among rural residents, only shorter daily duration of AT was association with lower prevalence of obesity (OR = 0.66, *P* < 0.05) and cholesterol disorder (OR = 0.73, *P* < 0.05). No significant difference was found among different age groups.

Logistic regression results also show that, after adjustment, risks of listed chronic diseases increased with weekly duration of AT. Differences on obesity, central obesity, and cholesterol disorder were statistically significant (see Table 5).

## 4. Discussion

Against the background of people increasingly using passive transport, as much as 49.6% of subjects from different groups of age, educational level, and household income level were walking and/or bicycling in their daily transport, which illustrates that walking and bicycling are still one of the main modes of transport for Jiangsu residents. Additionally, data of average frequency (5.3 days/week), daily duration (53.5 minutes), weekly duration (300.3 minutes) of AT, and higher percentages of active level of physical activity among AT respondents demonstrate that AT contributes much toward physical activity, which is consistent with existing evidence about active travel [16, 17].

Although it is widely believed that, from a societal point of view, the shift from car to walking and bicycling may have beneficial health effects due to increased levels of physical activity and decreased air pollution emissions [18–22], in this research, we found that having AT is related with higher prevalence of cholesterol disorder. We also found that, higher frequency, longer daily duration, and weekly duration of AT were associated with chronic diseases, such as obesity, central obesity, and cholesterol disorder, even after adjustments for age, gender, educational level, household income level, and overall physical activity level. To exclude the possible confounding effect of diet and life style, we analyzed the consumption amount of oil (gram/per capita/day within households) and daily sedentary time of each subject; results showed that AT respondents intake less oil (*d* = 0.9 gram, *P* < 0.01) and have shorter sedentary time (*d* = 22 minutes, *P* < 0.01) compared with non-AT respondents. Yet, we have no idea whether there is any other diet pattern or behavior among AT respondents which may affect the metabolism of fat.

Although there were some studies reported that walking or bicycling is not associated with benefit on health indices or body fitness before [23, 24], this finding runs counter to nearly all previous research, except that Hu et al. found that commuting or leisure-time physical activity was inversely related to mean BMI and the highest mean blood pressure in a cross-sectional survey [25]. Jiangsu Province of China is at a stage of fast economic development and transition. Thus, it is allocated increasingly more financial and material resources to the building of motor-vehicle-oriented transport infrastructure in the province. However, this consistently reduces space left for pedestrians and riders. It is supposed that the health effect of AT could be undermined by negative side effects of AT, such as more absorption of polluted air, and stress and tension from unsafe road conditions [26, 27]. In this study, we found that AT influences more health aspects among urban residents than among rural residents. Further information is needed concerning whether this is related to possible pressure from the more complex traffic situation in cities.

Although the underlying reason for this inverse correlation needs further study, this research highlights the need for the decision to promote AT to be discussed in depth from the health-related aspect. Jiangsu Province is a typical area with fast economic development and a big population; therefore, the research findings will be readily applicable to other similar areas. 

For limitations of this research, walking and bicycling were not distinguished, which may induce confoundation in the health effect, because it is reported that health effects of walking and bicycling are not identical for their different intensities [28]. Traditional bicycling and electric bicycling were measured together as well. At present, there is little evidence on the health effect difference between traditional and electric bicycling, though electric bikes shoot up and by far compose slightly less than half of the entire bicycle population in China [29]. Moreover, some of these differences are close, though these results are statistically different. Clinical meaning of these differences needs further discussion as well.

## 5. Conclusions

Walking and bicycling are still the main modes of transport for Jiangsu residents and they contribute much toward physical activity. The findings of this study did not support the notion that AT is beneficial to health at a population level. Further research is needed in determining the negative side effects of AT.

## Figures and Tables

**Table 1:** Characteristics of study participants (%).

	AT	Non-AT	Total
Gender*			
Male	41.72	48.82	45.30
Female	58.28	51.18	54.70
Residence			
Urban	35.45	35.97	35.71
Rural	64.55	64.03	64.29
Age*			
18–34	10.01	14.10	12.07
35–44	17.28	23.64	20.49
45–54	22.18	23.59	22.89
55–64	27.72	20.55	24.11
~65	22.80	18.12	20.44
Educational level*			
Low	51.15	44.71	47.90
Medium	42.22	49.50	45.89
High	6.63	5.79	6.20
Household income level*			
Low	41.67	35.16	38.42
Medium	32.82	34.84	33.83
High	25.51	30.01	27.75
Overall physical activity level*			
Inactive	7.42	21.15	13.28
Moderate	34.66	33.07	33.98
Active	57.92	45.78	52.74

*P < 0.01.

**Table 2:** Health indices/outcomes differences between AT and non-AT respondents.

	AT respondents			Non-AT respondents		
	Prevalence	95% CI		Prevalence	95% CI	
	/mean	Lower	Upper	/mean	Lower	Upper
Obesity (%)	13.88	12.83	14.93	13.40	12.38	14.43
Central obesity (%)**	42.95	41.44	44.45	40.79	39.31	42.27
Hypertension (%)*	50.97	49.45	52.49	47.34	45.84	48.85
Diabetes (%)	8.76	7.90	9.62	8.22	7.39	9.05
Cholesterol disorder (%)*	34.36	32.92	35.80	29.69	28.32	31.07
BMI (cm)	24.35	24.24	24.45	24.35	24.24	24.45
WC (cm)*	82.50	82.21	82.79	82.44	82.15	82.74
SBP (mmHg)*	139.21	138.53	139.88	137.66	136.98	138.34
DBP (mmHg)	84.83	84.49	85.18	84.92	84.58	85.27
FBG (mmol/L)	5.63	5.59	5.67	5.57	5.53	5.62
TC (mmol/L)*	4.37	4.33	4.40	4.49	4.46	4.52
HDL-C (mmol/L)*	1.32	1.31	1.33	1.38	1.36	1.39
LDL-C (mmol/L)	2.27	2.25	2.29	2.28	2.26	2.31
TG (mmol/L)	1.53	1.48	1.57	1.49	1.45	1.53

*P < 0.01;**P < 0.05.

**Table 3:** Health outcomes/indices and frequency and duration of AT.

	Frequency (days/week)			Daily duration (minutes/day)		
	1-2	3–6	7	<30	30–60	>60
Obesity (%)	13.63	12.85	14.58	10.70	14.53	15.12
Central obesity (%)	41.43	41.08	44.50	41.09	42.32	44.68
Hypertension (%)^∗†^	48.02	48.99	53.02	45.81	50.19	54.84
Diabetes (%)	7.61	7.21	10.03	7.34	9.50	8.86
Cholesterol disorder (%)^†^	31.38	33.81	35.55	29.66	35.01	36.50
BMI (cm)	24.11	24.31	24.44	24.06	24.43	24.44
WC (cm)*	81.96	82.07	82.92	81.67	82.59	82.91
SBP (mmHg)^†^	138.97	138.78	139.53	135.70	139.86	140.64
DBP (mmHg)	84.42	84.78	84.98	83.87	85.35	84.89
FBG (mmol/L)	5.55	5.53	5.72	5.60	5.62	5.66
TC (mmol/L)*	4.38	4.36	4.37	4.39	4.40	4.32
HDL-C (mmol/L)^†^	1.35	1.32	1.31	1.35	1.31	1.31
LDL-C (mmol/L)	2.22	2.25	2.30	2.26	2.30	2.25
TG (mmol/L)	1.44	1.42	1.61	1.48	1.55	1.53

*P < 0.05 between different groups of frequency; ^†^ *P* < 0.05 between different groups of daily duration.

**Table 4:** Logistic regression results with AT and prevalence of health outcomes.

	Category	OR	95% CI	
			Lower	Upper
Obesity				
AT or Non-AT		1.04	0.90	1.21
Frequency (days/week)^$^	1-2	0.98	0.73	1.30
	3–6	0.88	0.71	1.10
	7			
Daily duration (minutes/day)^#^	<30*	0.63	0.48	0.83
	30–60	0.92	0.75	1.14
	>60			

Central obesity				
AT or Non-AT		0.98	0.88	1.09
Frequency (days/week)	1-2*	0.81	0.66	1.00
	3–6	0.87	0.74	1.01
	7			
Daily duration (minutes/day)	<30*	0.82	0.68	0.99
	30–60	0.91	0.78	1.06
	>60			

Hypertension				
AT or Non-AT		0.98	0.88	1.10
Frequency (days/week)	1-2	0.86	0.70	1.07
	3–6	0.91	0.78	1.07
	7			
Daily duration (minutes/day)	<30	0.87	0.72	1.06
	30–60	0.93	0.79	1.10
	>60			

Diabetes				
AT or Non-AT		1.04	0.86	1.25
Frequency (days/week)	1-2	0.72	0.50	1.04
	3–6*	0.70	0.53	0.92
	7			
Daily duration (minutes/day)	<30	0.93	0.67	1.30
	30–60	1.11	0.85	1.45
	>60			

Cholesterol disorder				
AT or Non-AT		1.26	1.13	1.41
Frequency (days/week)	1-2	0.81	0.66	1.00
	3–6	0.91	0.78	1.06
	7			
Daily duration (minutes/day)	<30*	0.68	0.56	0.82
	30–60	0.91	0.78	1.06
	>60			

(1) All adjusted by age, gender, educational level, household income level, and overall physical activity level.

(2) Probability for entry 0.05, for removal 0.10.

(3) 0 for non-AT or nodisease, 1 for AT or disease.

(4)  ^$^Taking “7 days/week” as control; ^#^taking “>60 min/day” as control.

(5)  *P < 0.05.

**Table 5:** Logistic regression results with AT weekly duration and health outcomes.

Weekly duration (minutes/week)^$^	OR	95% CI	
		Lower	Upper
Obesity			
<105*	0.72	0.54	0.95
105–420*	0.79	0.63	0.99
>420			
Central obesity			
<105*	0.76	0.62	0.94
105–420	0.85	0.72	1
>420			
Hypertension			
<105	0.83	0.68	1.03
105–420	0.87	0.73	1.03
>420			
Diabetes			
<105	0.75	0.53	1.06
105–420	0.9	0.69	1.19
>420			
Cholesterol disorder			
<105*	0.64	0.52	0.79
105–420	0.86	0.73	1.01
>420			

(1) All adjusted by age, gender, educational level, household income level, and overall physical activity level.

(2) Probability for entry 0.05, for removal 0.10.

(3) 0 for non-AT or nodisease, 1 for AT or disease.

(4)  ^$^Taking “>420” as control.

(5)  *P < 0.05.
